# Hepatotoxicity of dietary supplements containing *Garcinia gummi-gutta* (L.) N. Robson

**DOI:** 10.1080/13880209.2025.2591467

**Published:** 2025-11-20

**Authors:** Richard B. van Breemen, Amy L. Roe, Nadeem Akhtar

**Affiliations:** ^a^ Department of Pharmaceutical Sciences, College of Pharmacy, Linus Pauling Institute, Oregon State University, Corvallis, OR, USA; ^b^ Procter & Gamble Healthcare, Cincinnati, OH, USA; ^c^ United States Pharmacopeia-India, Shamirpet, Hyderabad, India

**Keywords:** Garcinia gummi-gutta (L.) N. Robson, garcinia cambogia, hydroxycitric acid, hepatotoxicity, drug-induced liver injury, dietary supplements

## Abstract

**Background:**

Botanical dietary supplements derived from the fruit of the tree *Garcinia gummi-gutta* (L.) N. Robson (commonly known as *Garcinia cambogia* or *Garcinia*) are used to support weight loss but are increasingly linked to adverse events and case reports of liver injury.

**Objective:**

Clinical case reports of liver injury associated with *Garcinia* dietary supplements were reviewed that had prompted the United States Pharmacopeia (USP) to revise the USP *Garcinia* family of dietary ingredient monographs to include a cautionary statement regarding potential risk of liver damage.

**Methods:**

The terms ‘*Garcinia cambogia*,’ ‘*Garcinia gummi-gutta*,’ or ‘*Garcinia*’ were searched in multiple databases of adverse events. PubMed and Google Scholar were searched for peer-reviewed papers describing preclinical and clinical studies of *Garcinia* toxicity.

**Results:**

More than 200 adverse events of liver injury resulting from *Garcinia* consumption were identified. A total of 34 case reports of *Garcinia* hepatotoxicity indicated one death and nine liver transplants, with 17 cases receiving CIOMS/RUCAM scores that indicated possible to highly probable causality due to *Garcinia* dietary supplements. In one case, causality was confirmed by rechallenge with *Garcinia*.

**Discussion and Conclusions:**

*Garcinia* toxicity was consistent with drug-induced liver injury and included elevated serum liver enzymes alanine aminotransferase (ALT) and aspartate aminotransferase with a high ratio of ALT to alkaline phosphatase. Proposed mechanisms of toxicity include genetic predisposition to immune-mediated reactions involving the human leucocyte antigen HLA-B*35:01 allele, induction of hepatocyte oxidative stress and inflammation, and hepatocyte apoptosis caused by the active constituent, hydroxycitric acid, which inhibits mitochondria ATP-citrate lyase.

## Introduction

Native to South Asia and Southeast Asia, the fruit of the tree *Garcinia gummi-gutta* (L.) N. Robson (commonly known by its former botanical name *Garcinia cambogia*) has been used traditionally as a food, a food preservative, and in Ayurvedic medicine (Anilkumar et al. 2023). *Garcinia* powder and extract have also become popular in botanical dietary supplements marketed for weight loss (Heymsfield et al. 1998). The major chemical constituents are (–)-hydroxycitric acid (HCA) and its lactone, which are considered active and inactive, respectively. Hydroxycitric acid is present in the pericarp of the fruit at approximately 30% (w/w) but readily converts to the lactone. Commercial extracts of *Garcinia* often contain added calcium, sodium, or potassium to form the conjugate base, hydroxycitrate, and prevent formation of the lactone (Majeed et al. 1998).

The *United States Pharmacopeia–National Formulary* (*USP–NF*) defines *Garcinia* as the dried pericarp of the fruits of *G. gummi-gutta* (L.) N. Robson containing ≥12% (w/w) of the sum of hydroxycitric acid and the corresponding lactone. Similarly, *Garcinia* extracts are defined as being prepared using water, ethanol, or mixtures of these solvents, followed by stabilization of hydroxycitrate in the form of a calcium, potassium, magnesium, and/or sodium salt. The ratio of plant material to extract ranges from 5:1 to 10:1, and the dried extract should contain ≥40% hydroxycitrate (w/w). In commerce, *G. indica* and *G. atroviridis* are sometimes inadvertently substituted for *G. gummi-gutta*. All three species contain HCA. According to the Indian Council of Medical Research (Gupta 2005), the recommended daily intake of *Garcinia* is 2.4 g/day of dry powdered fruit rind, which is equivalent to *Garcinia* extract of 240–480 mg/day (depending on the plant-to-extract ratio).

The admission evaluation for *Garcinia* was initially conducted in January 2009 and subsequently updated in December 2017, in accordance with the *Guideline for the Admission of Dietary Supplement Ingredients to the USP–NF Monograph Development Process* (USP 2009). The results of the Admission Evaluation were published in the *USP Dietary Supplements Compendium.* Case reports in the literature about *Garcinia* and hepatotoxicity were noted during both the 2009 and 2017 USP reviews, but causality could not be clearly attributed due to limited information (United States Pharmacopeia 2024a, 2024b). The Dietary Supplement Admission Evaluation and Labeling Expert Committee continued monitoring clinical case reports, particularly those related to hepatotoxicity, and in 2024 recommended a cautionary labeling statement for potential liver toxicity. This decision prompted the Botanical Dietary Supplements & Herbal Medicines Expert Committee to revise the USP *Garcinia* family of dietary ingredient monographs to include a cautionary statement alerting consumers to the potential risk of liver damage (USP Botanical Dietary Supplements & Herbal Medicines 2024). This review describes the data suggesting that *Garcinia* might pose a risk to human health.

## Mechanism of action

The mechanism of action of *Garcinia* dietary supplements in promoting weight loss remains uncertain but is probably associated with its abundant constituent HCA. Han et al. (Han et al. 2022) reported that HCA and *Garcinia* extract inhibited lipogenesis in an adipocyte cell model (3T3-L1). In particular, HCA has been shown to inhibit conversion of mitochondria-derived citrate to acetyl-coenzyme A by ATP-citrate lyase, which is essential for the biosynthesis of fatty acids, cholesterol, and triglycerides (Soni et al. 2004). Selim et al. (2023) suggested that in situations where hepatocyte energy demands are high, HCA might inhibit ATP production from acetyl-CoA by mitochondria and create localized ischemia leading to apoptosis and hepatic necrosis.

## Toxicity in animal models and *in vitro*

In obesity-prone C57BL/6J male mice fed a high-fat diet, supplementation with *Garcinia* extract (1% w/w feed; 60% HCA) for 112 days resulted in hepatic fibrosis, inflammation, and oxidative stress (Kim et al. 2013). *Garcinia* supplementation increased serum levels of the liver enzymes alanine aminotransferase (ALT) and aspartate amino transferase (AST), caused hepatic collagen accumulation and lipid peroxidation, and increased mRNA levels of genes related to oxidative stress (superoxide dismutase and glutathione peroxidase) and inflammatory responses (tumor necrosis factor-α and monocyte chemoattractant protein-1). In contrast, Shara et al. (2004) administered *Garcinia* extract (5% w/w of feed intake; 60% HCA) by gavage to male and female Sprague-Dawley rats for 90 days and found no changes in hematology or clinical chemistry. These contradictory results were probably the result of species differences and differences in experimental design. For example, the study showing elevated liver enzymes and oxidative stress associated with *Garcinia* extract consumption used mice prone to develop obesity that were provided a high-fat diet, whereas the study finding no evidence of liver damage used healthy rats consuming a normal laboratory diet.

The National Toxicology Program carried out *in vitro* genotoxicity studies on a *Garcinia* extract using the Ames test with or without metabolic activation, and the results were negative under all conditions tested (National Toxicology Program, 2017). Lee and Lee (2007) reported that HCA extracted from *Garcinia* was negative in the Ames test and negative in an *in vitro* chromosomal aberration test. However, at the highest dose tested (12,500 μmol/kg), HCA was found to induce micronucleated polychromatic erythrocytes in mice which may be considered a weak mutagenic effect (Lee and Lee 2007).

No pharmacodynamic interactions of *Garcinia* with prescription drugs have been reported. However, an *in vitro* study indicated that *Garcinia* inhibits the cytochrome P450 enzyme CYP2B6 in a time-dependent manner and has the potential to cause pharmacokinetic interactions with CYP2B6 substrates *in vivo* (Yu et al. 2017). Some prescription drugs that are metabolized extensively by CYP2B6 include the antidepressant buproprion and the antiviral agent efavirenz.

## Human toxicity

### Clinical trials

Several clinical trials designed to evaluate the efficacy of *Garcinia* alone or in combination with other botanicals in promoting weight loss have included information on adverse events. Although these clinical trials reported no hepatoxicity, the number of participants and short duration of most of these investigations might have been insufficient to detect rare or delayed hepatic injuries. For example, in a clinical investigation to assess effects of consumption of *Garcinia* extract on sex hormones in 44 participants, consumption of *Garcinia* extract (1,667.3 mg/day, containing 1,000 mg/day HCA) for 12-weeks did not show significantly altered serum clinical chemistry or alteration in levels of the hormones testosterone, estrone, or estradiol (Hayamizu et al. 2008). In a 12-week, randomized, double-blind, placebo-controlled trial of weight loss, 42 participants consuming *Garcinia* ethanolic extract (3 g/day) reported no weight loss, no serious adverse effects, and only mild gastrointestinal discomfort (Heymsfield et al. 1998). Another 12-week randomized, double-blind, placebo-controlled clinical trial of 98 participants consuming a polyherbal product containing *Garcinia* also reported no weight loss or adverse events (Opala et al. 2006). A pharmacokinetics study involving 16 women each receiving a single dose of *Garcinia* extract (1,500 mg; containing 750 mg HCA) produced no changes in blood chemistry (Cruz et al. 2021). A prospective clinical trial without placebo control of 214 obese participants receiving extracts of *Garcinia* (500 mg twice per day; containing 52.4% HCA) and *Amorphophallus konjac* (500 mg twice per day; containing 94.9% glucomannan) for 6 months also reported no adverse effects (Maia-Landim et al. 2018).

However, in a prospective study conducted by the Drug Induced Liver Injury Network (DILIN) (Vuppalanchi et al. 2022), investigators identified 22 cases of drug-induced liver injury (DILI) associated with *Garcinia* among 1,987 patients from September 2004 through April 2018. Among these, five cases were attributed to *Garcinia* alone, 16 occurred in patients taking *Garcinia* in combination with green tea extract, and one patient consumed both *Garcinia* and Ashwagandha. The onset of liver injury ranged from 35 to 139 days after patients began taking *Garcinia*. Acute hepatitis with hepatocellular injury were accompanied by markedly elevated serum ALT and AST and modestly elevated ALP levels, which indicated hepatocellular disease. There was one death, and one patient required liver transplantation. The authors noted that DILI in patients consuming green tea extract showed symptoms similar to those of *Garcinia*. A major limitation of this study was lack of chemical analyses for all the herbal and dietary supplements used by the patients. The human leucocyte antigen HLA-B*35:01 allele was noted as occurring more often in the liver injury cases associated with *Garcinia* than with other botanical dietary supplements or conventional pharmaceuticals. An immune-mediated mechanism of injury was suggested due to possible association with the HLA-B*35:01 allele (Vuppalanchi et al. 2022).

Located on chromosome 6, the genes of the human leukocyte antigen (HLA) system encode cell-surface proteins responsible for regulation of the immune system (Choo 2007). The HLA-B*35:01 allele has been associated with autoimmune liver injury characterized by significantly increased AST and ALT, slightly elevated ALP, and moderate jaundice. In addition to *Garcinia* (Vuppalanchi et al. 2022), this pattern of liver injury associated with the HLA-B*35:01 allele has been reported for the drug terbinafine (Nicoletti et al. 2017) and the botanicals *Polygonum multiflorum* (Li et al. 2019), and *Camellia sinensis* (L.) Kuntze (green tea) (Vuppalanchi et al. 2022).

### Adverse events

The terms ‘*Garcinia cambogia*,’ ‘*Garcinia gummi-gutta*,’ or ‘*Garcinia*’ were searched in multiple databases of adverse events. Although more than 200 adverse events associated with *Garcinia* consumption have been reported in these databases, lack of information on the specific products including dosage, chemical composition, comorbidities, and attributes such as dechallenge or rechallenge, usually prevented determination of causation. Hepatotoxicity followed by central nervous system effects resembling serotonin syndrome were the most frequent adverse events.

In the U.S. Food and Drug Administration (FDA) Center for Food Safety and Applied Nutrition Adverse Event Reporting System for the period of January 2004–July 2025, 129 reports describe a wide range of adverse reactions to *Garcinia* (US FDA 2025). The most common adverse events were associated with hepatic injury (elevated serum hepatic enzymes, increased bilirubin, abdominal distension, and upper abdominal pain) followed by central nervous system disorders (depression, elevated mood, irritability, somnolence, aggression, anger, anxiety, and mania). Less frequent adverse events included constipation, nausea, diarrhea, headache, malaise, hypersensitivity, rash, increased blood pressure, increased blood clotting time, ocular icterus, myalgia, and pancreatitis. Most of these adverse events were associated with the consumption of *Garcinia* alone, while only 10 reported concomitant use of *Garcinia* with other dietary supplements or drugs. Two deaths were associated with *Garcinia* and were attributed to acute pancreatitis and atrial fibrillation, respectively (US FDA 2025).

The Canada Vigilance Adverse Reaction Online Database reported 123 adverse event reactions to *Garcinia* for the period of 01 January 1965–30 April 2025 (Health Canada, 2025). The reactions included nausea, vomiting, sinus tachycardia, abdominal pain, chest pain, pulmonary arterial hypertension, bradycardia, bundle branch block, ulcerative colitis, acute kidney injury, dehydration, diarrhea, or skin issues like rashes and ecchymosis. No reports were found for *Garcinia*-containing products in the Medicines and Healthcare Products Regulatory Agency (2025).

For the period 01 January 1971–29 July 2025, the Australian Therapeutic Goods Administration Database of Adverse Event Notifications, attributed two deaths and 71 adverse event reactions to *Garcinia* (TGA 2025). *Garcinia* alone was suspected in four cases, while the other reports involved combinations of *Garcinia* with other products. Most of these adverse events were related to hepatotoxicity (increased serum liver enzymes, upper abdominal pain, jaundice, and hepatitis) or central nervous system disorders resembling serotonin syndrome (anxiety, headache, and increased heart rate). Finally, the WHO Uppsala Monitoring Center database, VigiAccess, reported 75 adverse event reactions to *Garcinia* (UMC 2025). Among these adverse events, 9% (16) were due to hepatotoxicity, 12% (23) were attributed to nervous system disorders, and 15% (29). were related to gastrointestinal disorders.

### Case reports

**Figure 1.**
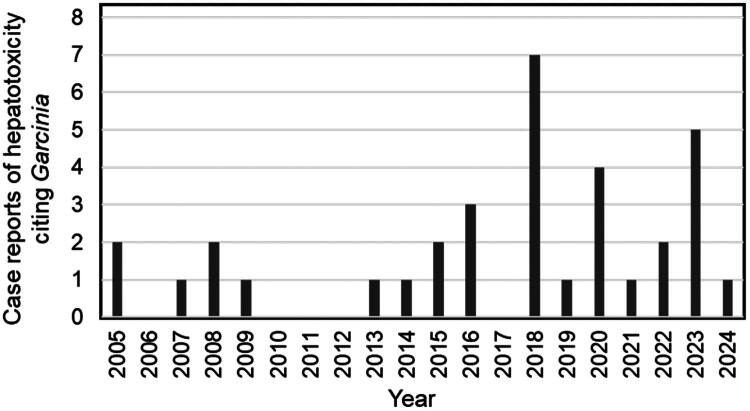
Annual trends in case reports of hepatotoxicity associated with *Garcinia.*

PubMed and Google Scholar were searched for peer-reviewed papers describing case reports of *Garcinia* toxicity, and 34 were identified. Compared with adverse events reports, case reports usually contain more details with respect to clinical diagnosis, dosage, description of *Garcina* product that was consumed, and causality. More females than males suffered liver injury linked to *Garcinia* use (26 out of 34 cases; 76%), perhaps because females use dietary supplements for weight loss more frequently than do males. Reports of liver injury due to *Garcinia* use have been increasing over the last 20 years (Figure 1). An average of one case of liver damage due to *Garcinia* was reported each year from 2005 until 2015, and subsequently, the rate has increased to an average of 2.4 case reports per year (Table 1).

**Table 1.** Case reports of hepatotoxicity associated with consumption of *Garcinia.*

Year	Age (years)	Gender	Length of Use	Dosage mg/day	Concomitant Drugs and Supplements	Diagnosis	CIOMS/ RUCAM^a^ Score	Outcome	Reference
2005	27	M	35		*Gymnema sylvestre* leaf extract, willow bark extract, glucomannan, green tea leaf extract, guarana extract (Hydroxycut)	Hepatocyte necrosis		Recovered	Stevens et al. (2005)
2005	30	M	5		*Gymnema sylvestre* leaf extract, willow bark extract, glucomannan, green tea leaf extract, guarana extract (Hydroxycut)	Cholestatic liver injury		Recovered	Stevens et al. (2005)
2007	45	F	8		Montelukast, paracetamol	Liver failure		Death	Actis et al. (2007)
2008	40	F	7		Levothyroxine, Hydroxycut combination supplement	DILI^b^		Recovered	Dara et al. (2008)
2008	33	F	14		Ortho-Novum contraceptive, Hydroxycut combination supplement	DILI		Recovered	Dara et al. (2008)
2009	25	M	21		*Gymnema sylvestre*, green tea extract, L-carnitine	DILI; liver failure		Liver transplant	McDonnell et al. (2009)
2013	48	F	28		Levothyroxine	DILI; liver failure	7	Liver transplant	Bessone et al. (2022)
2014	16	M	23		No	DILI	5	Recovered	Bessone et al. (2022)
2015	41	M	56		Hydroxycut combination supplement	DILI		Recovered	Araujo and Worman (2015)
2015	42	F	7		Hydralazine, hydrocodone, acetaminophen 3 days	DILI		Recovered	Melendez-Rosado et al. (2015)
2016	34	M	150	240 extract	No	DILI; liver failure		Liver transplant	Lunsford et al. (2016)
2016	52	F	25	936 extract 562 HCA^c^	Melatonin, dicyclomine	DILI; liver failure	7	Liver transplant	Corey et al. (2016)
2016	26	M	7		Whey protein powder	DILI; liver failure	6	Liver transplant	Smith et al. (2016)
2018	39	F	30	72 HCA	*Citrus aurantium*, Methyldopa, Domperidone, Omeprazole	Cholestatic hepatitis	6	Recovered	Crescioli et al. (2018)
2018	36	F	28		No	DILI	8	Recovered	Kothadia et al. (2018)
2018	57	F	30	2800 extract	Vitamins A and D	DILI	11	Recovered, rechallenged, recovered	Sharma et al. (2018)
2018	47	F	30	800 extract 400 HCA	Levothyroxine (100 μg/day), chromium (100 μg/day)	DILI	6	Recovered	Crescioli et al. (2018)
2018	52	F	30	400 extract 240 HCA	Green coffee (400 mg extract; 200 mg chlorogenic acid)	DILI	6	Recovered	Crescioli et al. (2018)
2018	61	F	60		Levothyroxine, *Ananas comosus*, *Ilex paraguariensis*	DILI	7	Recovered	Crescioli et al. (2018)
2018	33	F	30	1200 extract	Allium sativum (Garlic; 200 mg), *Trigonella foenum graecum* (fenugreek) (100 mg) × 2	DILI	8	Recovered	Philips and Augustine (2018)
2019	21	F	28	1400 extract	No	DILI	9	Recovered	Yousaf et al. (2019)
2020	26	F	210	1800 extract 900 HCA	Green tea extract, Veldt raisin, coffee	DILI; Liver failure	6	Liver transplant	Ferreira et al. (2020)
2020	64	F	15	1000–2000 extract	No	DILI	9	Recovered	Mas Ordeig and Bordón García (2020)
2020	39	F	35		No	DILI		Recovered	Al-Khazraji et al. (2020)
2020	22	F	90		Hydroxycut combination supplement	DILI	9	Recovered	Khetpal et al. (2020)
2021	54	F	60		4–6 standard alcohol drinks/day	DILI		Liver transplant	McCarthy et al. (2021)
2022	46	F	31		No	DILI	8	Recovered	Bessone et al. (2022)
2022	45	F	90		Banana leaf extract, brown seaweed extract	DILI		Recovered	Calaquian and Yau (2022)
2023	56	M	Unknown		α-Lipoic acid	DILI		Recovered	Le et al. (2023)
2023	47	F	3		No	Liver failure	2	Liver transplant	Selim et al. (2023)
2023	39	F	54		Green tea*, Gymnema silvestre*, piperine	Acute hepatitis		Recovered	Di Giacomo et al. (2023)
2023	42	F	Unknown	300 extract 180 HCA	Inulin 500 mg, green tea 20 mg	Jaundice, elevated ALT,^d^ AST,^e^ bilirubin		Recovered	Di Giacomo et al. (2023)
2023	57	F	30		Piperine, curcumin, Lasix, Eliquis, almarytm, Eutirox	Acute hepatitis		Recovered	Di Giacomo et al. (2023)
2024	65	F	90		No	DILI; liver failure	8	Liver transplant	Flerova et al. (2024)
*2005–2024*	*Mean* *41.4 (16–65)*	*M 23.5% (8/34)* *F 76.5% (26/34)*	*Mean 42.5* *(5–210)*	*Extract (240–2800)* *HCA (72–900)*	*Garcinia only, 26.5% (9/34)* *Garcinia plus concomitant drugs and supplements, 73.5% (25/34)*			*Death 2.9% (1/34)* *Liver transplant 26.5% (9/34)* *Recovered 70.6% (24/34)*	

Age, mean (SE), y: 41.4 (12.8).

Gender: M 23.5% (8/34); F 76.5% (26/34).

^a^ CIOMS: Council for International Organizations of Medical Sciences.

RUCAM: Roussel Uclaf Causality Assessment Method; Scoring: ≤ 0 excluded; 1–2 unlikely; 3–5 possible; 6–8 probable; > 8 definite or highly probable.

^b^ DILI: Drug-induced liver injury.

^c^ HCA: Hydroxycitric acid.

^d^ ALT: Alanine aminotransferase.

^e^ AST: Aspartate aminotransferase.

To the best of our knowledge, the first case report of a fatality associated with *Garcinia* hepatotoxicity was published in 2007 (Di Giacomo et al. 2023). A 45-year old obese female, who had been taking montelukast for asthma for 5 years, developed jaundice, elevated total bilirubin, markedly elevated AST and ALT 8 days after beginning treatment with two dietary supplements for weight control, one of which contained *Garcinia* (Table 1). The patient also reported taking paracetamol. The patient did not respond to treatment and died. Di Giacomo et al. (2023) speculated that this fatal hepatitis might be caused by interactions between *Garcinia* and montelukast.

We identified nine case reports of liver transplantation resulting from DILI associated with *Garcinia* consumption. In the first case report published in 2009 (McDonnell et al. 2009), a 25-year-old male developed fulminant liver failure requiring liver transplant after taking a combination dietary supplement for weight loss for 3 weeks that contained *Garcinia* (Table 1).

A second case of liver transplantation linked to *Garcinia* use was reported in 2013. A 48-year-old female taking levothyroxine for hypothyroidism developed jaundice, markedly increased AST and ALT, and slightly elevated ALP, 28 days after starting to take a daily dietary supplement for weight loss containing *Garcinia*. The Council for International Organizations of Medical Sciences (CIOMS)/Roussel Uclaf Causality Assessment Method (RUCAM) assessment score of 7 indicates probable causality due to *Garcinia.* The diagnosis was DILI, and the severity of liver damage necessitated liver transplantation (Table 1) (Bessone et al. 2022).

In 2016, a 34-year-old male developed highly elevated serum AST and ALT, mildly elevated ALP, and elevated total bilirubin after taking 240 mg of *Garcinia* extract per day for 5 months (Lunsford et al. 2016). The patient’s health continued to decline and required a liver transplant. Histopathologic examination of explanted liver tissue showed total hepatic necrosis with mixed inflammatory cell infiltrates resulting in a diagnosis of severe DILI (Table 1).

In another 2016 case report, a 52-year-old female developed jaundice and elevated serum AST, ALT, and total bilirubin (but not ALP) after taking 936 mg/day *Garcinia* for 25 days (Table 1). Computerized tomography showed shrunken nodular liver consistent with collapsing necrosis. The CIOMS/RUCAM score was 7, indicating that causality by *Garcinia* was probable. Tests for viral hepatitis, HIV, herpes viruses, and other viruses were negative, and the patient’s medical history was unremarkable, except for taking melatonin and dicyclomine for at least the previous year. Despite supportive management, the patient’s condition continued to deteriorate, and a surgical team performed a successful liver transplant approximately 50 days after the onset of symptoms (Corey et al. 2016).

In a 2016 case report from Australia, a 26-year-old male developed jaundice, fatigue, highly elevated serum AST and ALT, and moderately elevated ASP and bilirubin after consuming whey protein powder and a weight-loss supplement containing 70% *Garcinia* (Smith et al. 2016). The patient’s liver synthetic function deteriorated, and histopathology of a liver biopsy revealed submassive hepatic necrosis. After 2 months, the patient received a transplanted liver. The CIOMS/RUCAM score was 6, indicating that the dietary supplements probably caused DILI (Table 1).

A 2020 case of liver failure requiring liver transplantation was reported for a 26-year-old female with no medical history of liver disease (Table 1). For 7 months, the patient consumed three different dietary supplements daily for weight loss including 1,800 mg of *Garcinia* extract (containing 900 mg HCA), green tea extract, Veldt raisin, and *Coffea arabica* (Ferreira et al. 2020). Serum AST, ALT, total bilirubin but not ALP were significantly elevated, consistent with a diagnosis of DILI. The CIOMS/RUCAM causality score was 6, indicating a probable association with *Garcinia*.

In 2021, a 54-year-old female with no history of liver disease developed acute liver failure requiring transplantation after consuming *Garcinia* for 2 months (Table 1). The patient reported consuming 4–6 alcohol drinks daily. Assays for viral infection and autoantibodies were negative. Serum ALT, AST, bilirubin but not ALP were elevated. Explanted liver showed extensive necrosis with inflammatory cell infiltration, and the diagnosis was DILI secondary to *Garcinia* (McCarthy et al. 2021).

In 2023, a 47-year-old female with no significant medical history was diagnosed with ischemic liver injury and fulminant hepatic failure 3 days after starting to take a *Garcinia* dietary supplement (Selim et al. 2023). Serum AST and ALT were highly elevated, while ALP, total and direct bilirubin values were only slightly above normal values. However, the authors reported that the CIOMS/RUCAM score was only 2, indicating that DILI caused by *Garcinia* was unlikely (Table 1). The patient required a liver transplant, which was successful.

In a report of a liver transplant related to *Garcinia* consumption published in 2024, a 65-year-old woman with a history of breast cancer treatment developed obstructive jaundice after taking a *Garcinia* supplement for 3 months (Flerova et al. 2024). The patient developed hepatic encephalopathy, necessitating a liver transplant. The explanted liver exhibited multinucleated giant hepatocytes, hepatocytic necrosis, and substantial hepatocytic and canalicular cholestasis. Consistent with DILI, serum AST and ALT were markedly elevated, total and direct bilirubin were moderately elevated, and ALP was only slightly elevated. This case of severe hepatotoxicity received a CIOMS/RUCAM score of 8, indicating a probable connection with *Garcinia* use (Table 1).

The first case reports of hepatotoxicity involving *Garcinia* were published in 2005 and concerned two males consuming the same proprietary polyherbal formulation intended for weight loss (Stevens et al. 2005). Both patients recovered after discontinuing the dietary supplement. In addition to *Garcinia*, the product contained *Gymnema sylvestre* leaf extract, guarana extract, glucomannan, α-lipoic acid, willow bark extract, and green tea leaf extract (Table 1). In the first case, a 27-year-old male reported having taken the combination dietary supplement for 5 weeks. Besides jaundice, levels of serum AST and ALT were extremely elevated while ALP and bilirubin were only slightly above normal, leading to a diagnosis of hepatocyte necrosis consistent with DILI. In the second case, a 30-year-old male who had been taking an identical combination dietary supplement for 5 days developed jaundice, portal inflammation, slightly elevated levels of serum AST and ALT, and markedly elevated levels of ALP and bilirubin, resulting in a diagnosis of cholestatic liver injury (Table 1).

Only two out of 34 case reports of liver damage attributed to *Garcinia* dietary supplements have reported cholestatic hepatitis as the primary diagnosis (Table 1). In both cases, *Garcinia* was consumed in combination with other botanical dietary supplements, suggesting that other botanicals might have contributed to this form of liver damage. In the other case report of cholestatic hepatitis published in 2018 (Crescioli et al. 2018), a 39-year-old obese female on methyldopa, domperidone, and omeprazole developed jaundice, abdominal pain, elevated blood levels of hepatic enzymes, and direct bilirubin 1 month after beginning to take a dietary supplement containing extracts of *Citrus aurantium, Garcinia*, *Orthosiphon stamineus*, and *Griffonia simplicifolia*, and 15 days after adding a second dietary supplement containing extracts of *C. aurantium*, *Rhodiola rosea*, and *O. stamineus*. The diagnosis was acute cholestatic hepatitis, which resolved after discontinuation of all supplements and medications. The CIOMS/RUCAM score was 6, indicating a probable association with the botanical dietary supplements (Table 1).

In nine of the 34 case reports of hepatotoxicity associated with *Garcinia*, there were no concomitant uses of other botanicals, dietary supplements, alcohol, or medicines that might have contributed to liver injury (Table 1). This includes three of the nine cases resulting in liver transplants discussed above. Out of 18 case reports for which the authors assigned a CIOMS/RUCAM score, 17 rated causation due to use of dietary supplements as probable to definite (scores of 6–11), and seven of these cases identified *Garcinia* as the only supplement or medication used by the patient. The following six case reports concern patients who developed DILI after consuming *Garcinia* without any other supplements, alcohol, or medications and recovered without requiring liver transplantation.

A 16-year-old male with metabolic syndrome developed jaundice and elevated levels of AST and ALT 23 days in 2014 after using *Garcinia* but no other dietary supplements or medications (Bessone et al. 2022). Moderate DILI resolved after discontinuing *Garcinia.* The CIOMS/RUCAM-based causality assessment score of 5 suggested that the liver injury was possibly caused by *Garcinia* (Table 1).

In 2018, a 36-year-old female with no significant medical history developed jaundice and tender hepatomegaly after taking *Garcinia* but no other supplements for 1 month to lose weight. Laboratory tests indicated significantly elevated serum ALT, AST, and bilirubin, but only slightly higher than normal ALP, which is consistent with a diagnosis of DILI. The patient recovered within two weeks after discontinuation of *Garcinia*. The CIOMS/RUCAM score was 8 points, which indicated that *Garcinia* was the probable cause of DILI (Kothadia et al. 2018).

In a case report published in 2019, 21-year-old female with morbid obesity developed upper quadrant tenderness, significantly elevated ALT, AST, and moderately elevated ALP after using *Garcinia* but no other supplements or medications for 4 weeks in 2019. After discontinuing *Garcinia*, the patient recovered. The CIOMS/RUCAM score of 9 indicated that DILI was definitely caused by *Garcinia* (Yousaf et al. 2019).

In 2020, a 64-year-old female developed abdominal pain, and blood analysis indicated elevated direct bilirubin and liver enzymes AST, ALT, γ-glutamyltransferase (GGT), and ALP after consuming *Garcinia* (1,000–2,000 mg/day) for 15 days (Mas Ordeig and Bordón García 2020). The patient reported taking no medications or other dietary supplements during this time. The symptoms of DILI resolved after discontinuing the supplement, and the CIOMS/RUCAM score of 9 indicated highly probable or definite causality due to *Garcinia* (Table 1).

Also in 2020, there was a case report of a 39-year-old female who developed fatigue, dark colored urine, liver and scleral icterus, elevated serum liver enzymes, elevated total bilirubin, and mixed inflammatory cells in the portal tracts after consuming *Garcinia* for 5 weeks (Table 1). This patient was diagnosed with DILI and autoimmune hepatitis that resolved after discontinuation of *Garcinia* and immunosuppressive therapy (Al-Khazraji et al. 2020).

In a case report from 2022, a 46-year-old female presented with elevated serum liver enzymes due to mild liver damage after using *Garcinia* for 31 days (Bessone et al. 2022). The patient reported using no concomitant medications or herbal dietary supplements and had no comorbidities. After discontinuing *Garcinia*, the patient recovered in 30 days. The CIOMS/RUCAM score of 8 indicated that liver injury was probably caused by *Garcinia* (Table 1).

The remaining case reports concern patients diagnosed with DILI while taking *Garcinia* in combination with other botanical dietary supplements, vitamin supplements, or medicines. In two case reports of DILI associated with *Garcinia* from 2008 (Dara et al. 2008), a 40-year-old female taking levothyroxine for hypothyroidism developed acute hepatitis with moderately elevated AST and ALT, normal total bilirubin, and slightly elevated ALP, one week after using a combination dietary supplement containing *Garcinia.* In the other case, a 33-year-old female developed jaundice, fatigue, abdominal pain, elevated AST, ALT, and total bilirubin, but normal ALP after taking a dietary supplement containing *Garcinia* for 2 weeks to facilitate weight loss. Her only medication was an oral contraceptive. Both patients tested negative for hepatitis A, B, C, cytomegalovirus, and Epstein-Barr virus. After ceasing use of the *Garcinia* dietary supplements, both patients recovered (Table 1).

A 2015 case report described a 41-year-old male who developed fatigue and jaundice after consuming a dietary supplement containing *Garcinia* (Araujo and Worman 2015). Serum measurements showed extremely elevated serum AST, ALT, mildly elevated ALP, and moderately elevated total bilirubin 8.3 mg/dL. Assays for hepatitis, Epstein-Barr, and herpes simplex viruses were negative. The patient recovered completely after stopping consumption of the dietary supplement (Table 1).

In 2015, a 42-year-old female taking hydrazine for hypertension and a medical history of chronic kidney disease stage V, diabetes mellitus type 2, surgical resection of the gall bladder, and obesity developed upper abdominal pain after consuming only *Garcinia* for 1 week for weight loss (Melendez-Rosado et al. 2015). For 3 days, the patient had also been taking hydrocodone/acetaminophen (7.5/325 mg) every 4 to 6 h for back pain (Table 1). Lab tests indicated highly elevated serum ALT and AST, moderately elevated ALP and total bilirubin, but no indication of viral hepatitis. The patient recovered after stopping consumption of *Garcinia* and acetaminophen and receiving retreatment with *N*-acetylcysteine.

In a case report from 2018, a 57-year-old female with no previous history of liver disease developed acute hepatitis after taking vitamins A and D along with a high dose of *Garcinia* fruit rind extract (2,800 mg/day) for 1 month (Sharma et al. 2018). After discontinuing *Garcinia*, levels of ALT and AST returned to normal within 1 month. Blood levels of the liver enzymes ALT and AST were again elevated 6-months later after the patient resumed taking *Garcinia* to lose weight. This case was assigned a CIOMS/RUCAM score of 11, indicating that the cause of DILI was definitively *Garcinia* (Table 1). This was a unique case of causation being confirmed by rechallenging the patient with *Garcinia*.

In another case report from 2018, a 47-year-old female being treated with levothyroxine for hypothyroidism developed severe abdominal pain and elevated serum ALT, AST, GGT, ALP, and total bilirubin after taking a dietary supplement for 1 month for weight control (Crescioli et al. 2018). This product contained chromium and a high dose of *Garcinia* (800 mg/day; 400 mg/day HCA). The symptoms and blood parameters returned to normal after discontinuing the weight-loss supplement. The diagnosis was acute hepatitis with a CIOMS/RUCAM causality score of 6, indicating probable association with the dietary supplement (Table 1).

In 2018, 52-year-old female without previous serious medical problems was diagnosed with acute hepatitis 1 month after taking a dietary supplement containing *Garcinia* (400 mg extract; 240 mg HCA) and another supplement containing green coffee extract (400 mg extract; 200 mg chlorogenic acid) (Crescioli et al. 2018). Laboratory tests revealed a mild elevation of serum liver parameters AST, ALT, GGT, ALP, and total bilirubin. After discontinuing the weight-loss supplements, the acute hepatitis completely resolved without need of supplementary therapies. The CIOMS/RUCAM score of 6 indicated that the dietary supplements probably caused the liver injury (Table 1).

According to a 2018 case report, 2 months after taking a dietary supplement containing extracts of *Garcinia*, *Ananas comosus*, and *Ilex paraguariensis* in addition to levothyroxine, a 61-year-old female developed abdominal pain, jaundice, dark urine, markedly elevated blood levels of ALT and AST, and elevated total bilirubin, direct bilirubin, ALP, and GGT (Crescioli et al. 2018). The serum was negative for hepatitis viruses or autoantibodies. The diagnosis was DILI, which resolved over 4 weeks following cessation of the dietary supplement. The CIOMS/RUCAM score was 7, which indicates that the dietary supplement was the probable cause of the liver injury (Table 1).

Another case report from 2018 described a previously healthy 33-year-old female who developed nausea and elevated serum AST, ALT, and GGT but normal ALP and total and direct bilirubin level after using a multi-ingredient weight loss health supplement containing *Garcinia* (1,200 mg), *Allium sativum* (garlic), and *Trigonella foenum graecum* (fenugreek) for 1 month (Philips and Augustine 2018). The patient did not consume alcohol but did take calcium, vitamin D, and folic acid supplements daily. After discontinuing all supplements, the patient was treated with *N-*acetyl cysteine for one week and recovered. The CIOMS/RUCAM score for this case was 8, indicating that the supplements probably caused DILI (Table 1).

A 45-year-old female developed DILI after daily consuming a weight-loss supplement containing *Garcinia*, banana leaf extract, and brown seaweed extract for 3 months (Table 1) (Calaquian and Yau 2022). According to this 2022 case report, symptoms included mild hepatomegaly, hepatic necrosis with ductal proliferation, and elevated levels of serum ASP, ALT, total bilirubin, and direct bilirubin. The patient recovered after discontinuing *Garcinia* and receiving supportive care.

In a 2023 case report, a 56-year-old male with a medical history including sickle cell trait and use of the steroid oxymetholone 7 months earlier presented with generalized weakness, highly elevated serum AST and ALT and mildly elevated ALP and bilirubin after using the dietary supplements *Garcinia* and α-lipoic acid (Table 1). The patient recovered after discontinuing *Garcinia* and receiving supportive care (Le et al. 2023).

In 2023, Di Giacomo et al. (2023) reported three cases of DILI in women who consumed *Garcinia* in combination with other dietary supplements. In one case, a 39-year-old female consumed *Garcinia* (72 mg HCA/day) for 54 days along with green tea leaves, piperine, and *Gymnema silvestre* leaves. In the second case, a 57-year-old female consumed an unknown quantity of *Garcinia* in combination with curcumin and piperine along with the drugs Lasix, Eliquis, Eutirox, and almarytm. In the third case, a 42-year-old female consumed *Garcinia* (300 mg/day; containing 180 mg HCA) plus green tea extract (20 mg/day) and inulin (500 mg/day) for an unknown length of time (Table 1). All three recovered after stopping the supplements.

In a previous summary of 21 liver injury cases attributed to *Garcinia cambogia* consumption, Andueza et al. (2021) reported that seven cases involved the use of Hydroxycut^TM^ products. The US FDA issued a warning in 2009 on Hydroxycut^TM^ products related to hepatotoxicity due to 23 cases of liver damage, including one death and a liver transplant (US Food and Drug Administration 2009). Multiple products are marketed under the name Hydroxycut^TM^, all of which contain multiple botanicals but do not always include *Garcinia*. In the case studies summarized in Table 1, six reported that patients had consumed Hydroxycut^TM^ products containing *Garcinia*.

## Concluding remarks

Due to increasing numbers of adverse events and case reports of liver injury associated with the use of *Garcinia* dietary supplements, the Dietary Supplement Admission Evaluation and Labeling Expert Committee of the USP decided to introduce a cautionary labeling statement that reads, ‘*Dosage forms prepared with this article should bear the following statement: Do not use if you have a liver problem; discontinue use and consult a healthcare practitioner if you develop symptoms such as abdominal pain, dark urine, or jaundice (yellowing of the skin or eyes*).’ This committee continues to monitor the literature for any emerging adverse event reports along with causality assessment for *Garcinia* species.

Because weight loss products like *Garcinia* are among the most frequently adulterated or fraudulent dietary supplements in the United States (White 2018), some of the adverse events attributed to *Garcinia* might be due to additives not disclosed on the product labels. Another possible cause of hepatotoxicity involving *Garcinia* might be the simultaneous consumption of other botanicals or pharmaceutical agents with hepatoxic effects alone or in combination. Despite causing liver injury through several possible mechanisms of action, *Garcinia* hepatotoxicity remains rare and might be related to genetic variability that contributes to susceptability (Li et al. 2019).

## Data Availability

All raw data from original research papers and on-line databases are summarized in this review article, and the data sources are cited in the Reference section.
